# Understanding the Wellbeing Effects of a Community Music Program for People With Disabilities: A Mixed Methods, Person-Centered Study

**DOI:** 10.3389/fpsyg.2020.588734

**Published:** 2020-12-14

**Authors:** Una M. MacGlone, Joy Vamvakaris, Graeme B. Wilson, Raymond A. R. MacDonald

**Affiliations:** Reid School of Music, Edinburgh College of Art, University of Edinburgh, Edinburgh, United Kingdom

**Keywords:** community music, person-centered research, mixed methods, impact, individuals with disabilities, wellbeing

## Abstract

People with disabilities face inequalities in mental wellbeing, for which social exclusion is a contributing factor. Musical activities offer a promising but complex intervention, making impacts on a population with highly varied characteristics and needs challenging to capture. This paper reports on a mixed methods, person-centered study investigating a community music intervention for such a population. Three groups of adult service users with varied disabilities (either physical, learning, or both), took part in weekly music workshops in different locations. Music staff, housing and resource center staff, as well as participants and members of their families, took part in semi-structured interviews. A quantitative measure administered by service staff was used to rate service users’ social development. Two lay researchers, both individuals with a disability contributed to all aspects of the study. Interviews were analyzed through thematic analysis. Improvements in individuals’ self-expression, confidence, mood, and social skills were consistent with previous findings. Differences in effect between centers included: Group 1, some of whom had previous experience of workshops, showed an improvement in musical skills; Group 2 showed a mixed response, some participated with enthusiasm but others chose art activities over music workshops; Group 3 had lasting positive impact, this group had very limited opportunities for music due to their rural location. Quantitative analysis showed significant increase over all groups in communication, interaction with others, and joint attention. The intervention was beneficial for participants in separate locations in similar ways, but also highlighted that context and prior experience mediated effects in distinct ways. The lay researchers enhanced the qualitative analysis by emphasizing (1) the importance of recognizing participants’ self-expression in non-verbal modes of communication and (2) the importance of having music staff with a disability to provide a positive role model. This paper proposes that mixed methods person-centered research is the most suitable approach to capture and understand the multiple and varied effects of this complex intervention for a diverse group of participants.

## Introduction

Tackling social isolation is a priority in addressing the widespread inequalities experienced by people with disabilities. In Scotland, for example, mental wellbeing is lower among adults living with a limiting long-term health condition than in those living with no long-term health condition (Scottish Government, 2020). Isolation due to social exclusion is a key contributing factor to such mental health impacts, and therefore a crucial area for investigation in disability research (Barusch et al., 1999; De Jong Gierveld et al., 2006; Hawthorne, 2008; Honey et al., 2011; Kerr et al., 2012; Cacioppo and Cacioppo, 2014). Addressing social exclusion has therefore been identified as a way of improving the mental health of people with a disability (Honey et al., 2011; Scottish Government, 2016). Creative activity is associated with better wellbeing, and participation in cultural activities represents a positive coping strategy for people with disabilities who also have mental health issues (Kerr et al., 2012; Leadbetter and O’Connor, 2013; All-Party Parliamentary Group on Arts and Health and Wellbeing, 2017). The United Nations Convention on the Rights of Persons with Disabilities (CRPD) recognizes rights to participation in cultural life as important both for individuals with a disability and the societies in which they live, and access to creative learning opportunities as key for the development of the self, which can also positively impact their families and wider social circles. However, rates of cultural participation are lower among adults with a disability than among those without. (Scottish Government, 2019). Increasing access to cultural activities for such individuals is therefore a priority to achieve better social integration and mental health.

Participation in music in particular can address these priorities, through music therapy or community music. There are many approaches to and definitions of music therapy but key to all is the therapeutic relationship between a qualified music therapist and client, where music is used to achieve beneficial effects (MacDonald et al., 2012). These can be psychological, emotional cognitive, physical, communicative or social depending on the specific needs of the client (Geretsegger et al., 2014). Community music interventions are distinct as they may have positive therapeutic outcomes, but these are secondary to the realization of an enjoyable, sociable experience for participants (MacDonald et al., 2012). Making music with other people offers an enjoyable experience of profound and meaningful interaction for individuals with difficulties communicating verbally, building their social confidence and networks, and thus reducing isolation (Geretsegger et al., 2014; MacDonald and Wilson, 2014; Cobbett, 2016; Wheeler and Murphy, 2016). Performing music that has been personally devised or decided on is a powerful channel of creative expression that can radically change self-perceptions of disadvantaged individuals (MacDonald and Miell, 2002). A recent study of an inclusive workshop program for a group of individuals with disabilities, where participants had agency in composing with music staff and over the nature of their music making, found positive impacts (Wilson and MacDonald, 2019). It was observed that participation could increase their self-confidence, improve mood, engender creative agency, provide another channel of communication to aid interactions, and provide an absorbing purposeful activity. However, people with disabilities and the challenges they face are enormously diverse, not just in terms of the nature and level of their disability, but in demographic factors such as age or geographic location. The social context of participation in arts and health research also comprises multiple elements with influences likely to vary depending on settings, participant groups, goals, and health conditions (Clift et al., 2009). The complexity of challenges and settings is thus an essential consideration for any research into the impacts of an intervention for individuals with disabilities. For example, context sensitivity is stressed as fundamental to understanding the engagement and development of any person with an Autistic Spectrum Condition (ASC) (Bushell, 2017). In particular, impacts on personal qualities and wellbeing should be understood in relation to improvements to social interactions (Bishop, 1998).

It is also vital that any research on the experiences of marginalized groups attends to their unique experiences and perspectives. Four research principles were proposed to recognize and accommodate the needs of the individual in Jacobs et al. (2017): *connectivity* recognizes that researchers should accommodate other viewpoints*; attentiveness and dialog* require mindfulness of context and the implications of researcher’s co-presence; *empowerment and participation* afford participants agency in their choices; and *critical reflexivity* necessitates examining how power relationships are cultivated and sustained and who benefits from them.

This is especially pertinent in research with individuals with a disability. However, studies of music interventions for this population are methodologically heterogeneous (Moore and Goodley, 1999). The purpose of music interventions has often been to address specific elements of participants’ disabilities. For example, in music therapy research with individuals with Autistic Spectrum conditions (hereafter referred to as ASC), social impact is often measured through validated scales to investigate improvements in aspects of communication (Wetherby, 2006). For example, Bishop (1998) assessor-blinded parallel group RCT of a music therapy intervention with children with ASC used the CCC-2 checklist (Sharda et al., 2018) to assess improvements in social communication, pragmatics, inappropriate initiations, social relations and interests; and a family quality of life measure (Park et al., 2003) to assess improvements in family interaction, cohesion and coping. In community music research, broader aspects of social impact are sought. One influential study defined these in terms of ongoing effects on “personal development, social cohesion, community empowerment and self-determination, local image and identity, imagination and vision, health, and well-being” (Landry et al., 1995. p9). These in turn informed the categories of social impact reported in influential research by Matarasso (1997); however, Matarasso’s work has been criticized for developing *a priori* categories based on Landry et al., rather than seeking to understand subjective experiences of impact on participants’ wellbeing (Merli, 2002). Defining and evaluating the effects of community music workshops therefore presents distinct methodological challenges.

Research into the impacts of community music is scarce (Wilson and MacDonald, 2019) and there is a particular knowledge gap around how different settings are influential. Most individuals with a disability in Scotland attend community day centers who cater for groups who have different conditions and needs (Scottish Government, 2016). Community settings differ from therapeutic settings, because the focus is on the individual’s needs rather than addressing aspects of their condition. Another key factor is that those with a disability may not have a specific or fully accurate diagnosis (Fidler and Jameson, 2008). Because of this, mixed methods are an appropriate choice for community music research on a population with highly varied characteristics, as impacts and needs are challenging to capture. Effects may manifest in different ways, or not at all for participants, as interaction with music is a highly personal process (MacDonald et al., 2012). Therefore, gathering evidence of participants’ actions in music workshops is valuable, but understanding how they are in day centers and in their homes gives a fuller picture of potential benefits; and interviewing a range of key adults in participants’ lives, as well as themselves, can enrich insight into the person and their experiences. Researchers can use assessments developed specifically for the participants in an intervention (Grocke et al., 2014). In keeping with person-centered research, capturing and understanding potential effects from the position of the participant may present particular practical challenges, for example when researching a group comprised of people with different disabilities (i.e., physical, cognitive, or both) (Daykin, 2012). Social inequalities including unequal access to music and other arts may result in different experiences of participation (Daykin, 2012). Improving our understanding of how the effects of community music for individuals with disabilities can be fully captured and understood for different groups will allow any benefits to be made accessible to this population as a whole, and reduce the wider inequalities in health that they face.

This paper reports on the second stage of a recent research project in Scotland to address these gaps in knowledge. A community music intervention for young adults with learning disabilities that had shown positive impacts (Wilson and MacDonald, 2019) was scaled up in a second stage, with the aims of providing more detailed quantitative and qualitative evidence of health and wellbeing impacts, and showing how these might be transferrable to other disadvantaged groups. This paper focuses on findings related to these impacts, and addresses the following questions:

(1) Do the effects of community music workshops vary for different groups of adults with disabilities, and if so, how?
(2) How can qualitative, quantitative and participatory methods be combined to evaluate the range of impacts of community music programs?
(3) What potential do community music programs have to benefit a broad range of people with disabilities?

## Materials and Methods

### Design

Mixed methods were utilized with the purpose of complementarity, which “seeks confirmation, elaboration, enhancement, illustration, and clarification of the results from one method with the results from the other method” (Greene et al., 1989, p 259). Research design was *concurrent* (Creswell and Creswell, 2017) whereby the qualitative and quantitative elements are gathered simultaneously and their results compared. This study is defined as qualitatively driven (Johnson et al., 2007) because it privileges its main method (“QUAL”) in exploring and systematizing varied experiences across a field, while the additional method (“quant”) summates impacts at the field level.

### Participants

Group 1: Seventeen (13 male and 4 female) participants were recruited via three separate resource centers from an accessible rural location in Central Scotland. Ages ranged from 18 – 53. Group 2: Sixteen (12 male and 4 female) participants were recruited via two separate resource centers in an urban location in Central Scotland. Ages ranged from 30 to 73. Group 3: Twelve (8 male and 4 female) participants were recruited via one resource center in a rural location in Southern Scotland. Ages ranged from 22 to 67.

Within all groups, participants’ disabilities ranged from mild to profound and complex, and their levels of independence ranged from requiring constant one-to-one care to living alone in sheltered accommodation. More specific information about the nature of participants’ disabilities was not officially disclosed by the resource centers. This policy is guided by the recognition that the medical and personal information of a person with a disability is private to that individual (United Nations, 2006). Ethnicity data were not gathered for the same reason.

### Intervention

Workshops delivered followed the format, content and objectives of these detailed in Wilson and MacDonald (2019). In each workshop three musicians facilitated the session; one expert (over 20 years of experience); one trainee (≈1 years experience) and one musician with a disability (≈10 years of experience). The expert led most of the activities but as the workshop program progressed, the other facilitators led increasingly more activities. Activities comprised a combination of warm-up songs, singing or drumming along to African chants, folk music and pop tunes, played on instruments from BoomWhackers (pitched plastic pipes) to keyboards. Sessions ended with a closing “goodbye song.”

Activities were tailored for each of the groups above based on preferences expressed through the participatory process of the workshops. Two examples of these are: (1) inclusive activities which were differentiated. For example, participants could drum, sing, play adapted guitars, or keyboards according to their preference and (2) the flexible structure, whereby space was given to hear service users’ thoughts both during and between activities. Choosing the next song was often based on views from the group.

Many of Group 1 had participated in Stage 1 (see Wilson and MacDonald, 2019) so had existing knowledge and musical skills to build on. With music staff, the group jointly composed original song lyrics and had input into song structures. They also built a repertoire of pop cover songs and folk arrangements. This group performed at an end of project live showcase for family, center staff, the public and invited guests. The event was also live streamed on Facebook (≈500 views) and the project’s webpage.

Group 2 had not participated in Stage 1, however, five participants had previously attended workshops run by other disability music organizations in the city. They contributed song lyrics and suggested narrative directions of songs. Two participants requested songs they wanted the music workers to play, so they could perform them for the rest of the group.

Group 3 had not participated in Stage 1 and received 10 workshops from August-October, rather than the initially planned 20. This was due to the rural center that was first identified being unable to recruit sufficient attendees. Group 3 had very limited previous access to music apart from singing or playing percussion along with recorded music in sessions led by non-specialists. Outwith the music sessions, participants asked staff if they could sing workshop songs. Toward the end of the project, the center purchased a set of BoomWackers so they could continue making music in ways introduced to them during the workshops.

### Materials

In-depth interview topic guides were devised to gather information about particular difficulties faced by participants, their levels of confidence and sociability, aims for their personal growth in both center and home contexts, views on the workshops and any impacts or issues they perceived; and at the final interview, views on whether these impacts were still appreciated some weeks after the program. Interviews with music staff delivering the workshops gathered their perceptions of any changes in the participants’ musicianship, sociability and any other perceived impacts.

Quantitative data comprised a survey developed for service staff to assess participants’ social development. Participants were scored in five key areas of social development on a 5-point scale from very limited to very good. Paired-samples *t*-tests were conducted to compare scores in five key areas of social development before and after the workshop program. These were: *joint attention*, (I am sharing my focus with others, e.g., listening to music and sharing an activity); *communication* (I am making my needs known and responding to the communication of others); *interaction with others* (I interact with others by looking at, signing with, talking to others); *social awareness* (I am more aware of the people around me, e.g., look at them more frequently, sit with them) and *self-expression* (I am able to let others know what I am thinking/feeling).

Two lay researchers, both individuals with a disability, were recruited to take detailed observational notes on how they perceived participants engaged musically and socially in workshops for Groups 1 and 2 and to contribute to analysis.

### Procedure

The programs of music workshops were delivered by a registered charity highly experienced in this work (Limelight Music). Groups 1 and 2 received 10 workshops from April to July 2018; all three centers then received 10 workshops between August and October 2018. Workshops were weekly, with occasional breaks due to day center closures for holidays and a summer holiday of 5 weeks. Sessions were for 2 h with a 15-min break. Individuals who attended all sessions from Groups 1 and 2 received 35 h over 28 weeks, and those from Group 3 experienced 17.5 h of workshops over 12 weeks. All workshops took place in accessible rehearsal spaces, and encouraged continued social interaction between participants at breaktimes. In Group 2, six participants dropped out of the workshop program; all were given the choice to attend at the beginning of every workshop and chose to take part in art and crafts activities instead.

Interviews were gathered at three timepoints: baseline; post-program; and at five-week follow-up. Interviews were sought with service staff for each participant (center life interview) and either the participant, a family member, or key housing staff for participants (home life interview). Interviews were held in centers or in homes as convenient for participants. At baseline, 44 center life, 31 home life, and three music staff interviews were gathered. Post-program, 38 center life; 17 home life, and four music staff interviews were gathered. At five-week follow-up, 38 center life and 18 home life interviews were gathered.

The survey was administered by service staff at the program midpoint in early August; post-program; and at five-week follow-up. Table 1 shows numbers for both interviews and surveys achieved at each wave. For missing interviews, researchers contacted people three times to arrange alternatives. For missing surveys, each center was phoned three times and emailed twice for follow up, but had no response. The total number of surveys collected are as follows: at baseline, 31; at post-program, 27; and at five-week follow up, four surveys.

**TABLE 1:** Data collection table.

Time point	Baseline	Post-program	5-week follow up
**Group and data type**			
Group 1 interviews (16 at baseline, 1 participant joined after workshops commenced)	16 center life 11 home life	17 center life 11 home life	17 center life 7 home life
Group 2 interviews (16 at baseline, 6 participants left 3 weeks into program)	16 center life 10 home life	10 center life 3 home life	10 center life 3 home life
Group 3 interviews (12 participants at baseline. 1 left the country 2 weeks in to the program)	12 center life 10 home life	11 center life 3 home life	11 center life 8 home life
Center and home life interview totals	44 center life 31 home life	38 center life 17 home life	38 center life 18 home life
Music staff interviews (only to be gathered at baseline and post-program)	3	4	–
Music staff totals	3 music staff	4 music staff	–
Group 1 surveys	17	13	4
Group 2 surveys	3	3	0
Group 3 surveys	11	11	0
Survey totals	31	27	4

### Analysis

Interviews were analyzed using thematic analysis (Braun and Clarke, 2013). Transcripts were repeatedly read and then coded by one researcher for initial themes and discussed regularly by the research team to resolve divergent instances and arrive at a consistent coding of themes. Themes were patterns of interest noted in any or all interview types (i.e., participants, parents, service staff, housing staff, music staff, and lay researchers) and considered in relation to the whole data set. These were reviewed in two focus groups, one with the project advisory group and one with the two lay researchers, to ensure a consistent understanding between research team and the population being researched. The advisory group comprised service users who were not participating in workshops, the lay researchers, service staff from non-participating centers and an experienced community musician. The lay researchers also commented on a draft of this paper. Following the principles of person-centered research mentioned earlier from Jacobs et al. (2017), the lay researchers were asked if they wanted to be named in this paper and both chose to be identified^1^. Two overarching categories structure the results reported here: (i) common impacts which were described in all three groups and (ii) contextual impacts, which were themes distinct between groups or unique to one.

The quantitative data collected was analyzed using IBM SPSS V25 statistical software (IBM Corp, 2017). Through descriptive statistics and paired-sample *t*-tests, the analysis focused on identifying statistically significant change in reported social development aspects, looking at scores before and after the workshop program.

### Mixed Methods Analysis

**FIGURE 1:**
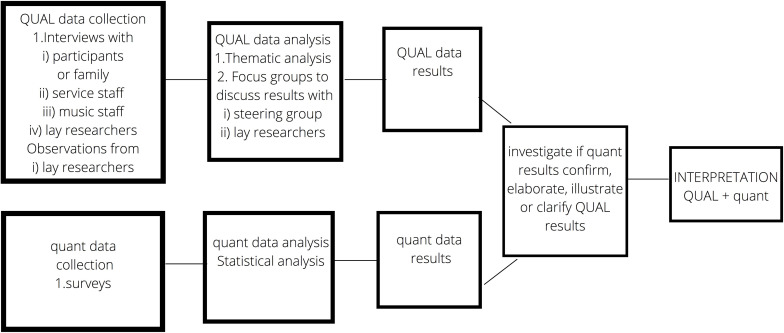
Mixed methods design.

Figure 1 shows the separate stages of data collection, both QUAL and quant analysis and mixing of results.

In complementarity mixed methods research, data are initially analyzed separately following the chosen methodological approaches (as described above) (see Greene et al., 1989). Following this, data were compared to address research question 1 and where the data related to the same theme, the datasets were compared. The next stage considered how results may converge. As this research is qualitatively led, quantitative results were viewed as providing extra perspective to the identified themes. The convergence of the data was then considered and the data were labeled based on the relationship of quant to QUAL. Possible labels relate to whether or if data clearly *confirms* or *contradicts* one another; is *mixed*, with both confirmatory and contradictory elements, or *enhances*, where extra understanding and depth is added to the themes.

Quantitative results were only topically relevant to the first category, common impacts and not to the second, contextual impacts.

### Ethics Approval Statement

Informed consent for research participation was gathered in advance from participants, or from an identified person with responsibility if they lacked capacity for this. To inform potential interviewees, accessible printed or recorded information sheets were prepared detailing the voluntary, confidential, and anonymous nature of participation and how data would be used.

This research received ethical approval from the Edinburgh College of Art Research Ethics Sub-Committee, University of Edinburgh, which includes consideration of any risk of disadvantaging individuals through the research.

## Results

Two overarching categories were identified, common impacts and contextual impacts. Common impacts were consistently noted in all types of interviewees’ accounts and in all of the groups; contextual impacts were mutually exclusive to each location. The category of common impacts was supported by relevant quantitative results and mixed methods analysis. The sections that comprise results are: common impacts, contextual impacts, quantitative results, and mixed methods analysis.

### Common Impacts

Improvements in individuals’ self-expression, confidence, mood, and social skills were attributed to workshop participation at all three settings.

#### Self-Expression

Participants’ emotions were recognized and accommodated through music staff altering existing songs or creating new ones. An example of a new song was “I feel alone” which explored feelings of, and coping with loneliness. The act of playing or singing was also seen to be a separate channel of expression. For example:

He’s actually playing the instruments, joining in and listening to music, songs being sung, and expressing himself through singing, which I have to say wasn’t something he did that often until he was actually working with the group (Service staff/G1/7)^2^.

An increase in verbal expression from some service users during the workshops was also noted: “he can speak freely (in workshops) as well” (Service staff/G2/4). Lay researcher KB commented that through the workshops participants were able to express their own “voice.” KB defined “voice” as a form of self-expression that could be “non-traditional” (i.e., non-verbal) in how it was communicated. For example, participants could sing, speak, play instruments, but also express their “voice” through increased eye contact with others, bodily movement or gesture.

#### Confidence

Staff and parents described participants having increased belief in themselves and their choices, pointing to them “speaking up” (Service staff/G1/2) in workshops or being more likely to make decisions for themselves outwith music sessions. For instance, one participant chose to sit next to different people in the workshops after a period of always choosing to sit between the same two service users.

Participants themselves talked about developing confidence, for example in their feelings about going into the sessions:

Well, the first day I was a bit nervous because I wasn’t sure what I was going into. I was like “what is this?” but as the weeks went on I’m confident enough to go to the workshops now (Participant/G1/1).

Developments in self-efficacy were also described in the everyday lives of service users:

Certainly in the last couple, two or three (months), I would say there’s been even more of a positive change in Cassie’s life. She’s even involved in making more decisions, she’s been very vocal about things that she wants to do and places she wants to go (Residential staff/G3/1).

For Cassie, her ability to be more “vocal” gave her greater agency to improve her experiences in a way which was meaningful to her. The staff saw her growth of confidence at the workshops as transferable. This was also demonstrated by three individuals (from Group 1) who took part in a government-led focus group to gather young adults’ opinions about key issues for them^3^ :

I’m not sure how a year ago the girls would have got on going into an environment with young folks… who very much know each other. I’m not sure how much they would have spoken and contributed. Whereas the progression, I think they’ve went into it with more confidence than they would have done before. I think that’s four meetings that they’ve been part of, they’re absolutely in the mix now. the girls are doing a great job. They’re raised loads of points about different things about inclusivity (Service staff/G1/9).

By making an effective contribution to this focus group, the three participants exercised newfound skills of listening, synthesizing information and being able to put views across on behalf of young adults with a disability.

#### Mood

Staff in all groups noted increases in happiness and relaxation among participants both during and after the workshops. Enjoyment was seen to be crucial in this:

I just enjoy the workshops, it’s good fun, a good laugh. It’s a good social activity (Participant/G1/3).

Interviewer: How do you feel after a workshop? Participant: Happy. That’s the overwhelming feeling is happy (Participant/G1/2).

It seems to be making him feel relaxed enough to be more open, rather than just being an observer, he’s obviously joining in (Service Staff/G3/3).

Shortly before Stage 2, staff in one of the Group 1 centers were researching strategies to target absenteeism and low mood through creating rewarding, meaningful activities. However, after the workshops started, those taking part in the workshops were seen to have improved past the need for extra intervention at their center. Staff also saw the music sessions as having “a calming influence” on anxious participants. Some non-verbal participants were observed displaying fewer compulsive behaviors associated with their anxiety, such as rubbing their head or chewing clothes. One staff member thought music helped a service user prone to frustration and anger because:

it kind of gets his mind off things. It looks… like he partially loses himself in the music; it’s a bit of escapism (Service staff/G2/1).

The workshops became an important part of the week, parents and staff reporting the participants’ positive anticipation the night before, or morning of the workshops.

#### Social Skills

Participants were described by others as becoming more inclined to meet with new people or people they did not know so well, and as more likely to enter situations where they did not know people. In the following extract a staff member describes how he understood a service user becoming more socially engaged through workshop participation:

I felt she (Violet) was just getting used to looking around the room … in the session and enjoying it. She became a lot more expressive, maybe shaking maracas and joining in… But she’d always arrive in a shell, and then blossom, if you like, by the end of it (Service staff/G1/5).

For this staff member, the first observable changes were in Violet’s gaze interactions. The workshops provided a space for Violet to interact with others at a pace that was suitable for her. She was able show this interaction though playing music, “joining in” when she chose. Another participant explained herself how her gaze shifted toward others as she felt more sociable:

When I first started I wasn’t looking at anybody, and I was just looking at the ceiling, but now I’ve stopped doing that, and talking to other people instead of Jackie (Participant/G1/1).

The expansion of her social circle was an important change for this individual. For others, this was as a motivating factor to attend more workshops, for example:

I think it would change my life a bit to come and do another music thing. Like I’m doing the dance group as well on a Wednesday and I feel like the Monday’s a good day for me to come and meet new people (Participant/G1/2).

This participant appreciates a potential gain from getting to know people at workshops so far, but also sees further possible benefits: “I think it would change my life.”

#### Summary of Common Impacts

Self-expression, confidence and mood were described through change in either individuals’ behavior, self-perception, or wellbeing. They can be seen as first affecting individuals on a personal level, whereas social skills developed through change in individuals’ communications and interactions with others. These effects are linked, as increased capacity to speak up (confidence) can result in enhanced self-expression. Another interpretation is that successful self-expression can give individuals a growing belief in themselves as competent communicators, which in itself leads to greater confidence. A combination of both of these qualities contributes to participants’ sociability, which can be understood as the ways their confidence and self-expression are enacted with others.

The next section reports on impacts for particular contexts.

### Contextual Impacts

Not all impacts from the workshops were observed in all three groups. This section presents distinct impacts found in each group.

#### Musical Skills

An improvement in both, or either, technical and musical skills was noted by service users and key stakeholders for Group 1 only. Music staff described progress in the following ways:

So, you can see the development there. And with everybody, everybody’s exerting more pressure, so things are becoming more physical as well as more musical and more social (Music staff/1).

An important stage in learning string instruments is when a student can effectively push the strings down to create a good, clear tone without buzzing. Music staff can appreciate the change in sound that the participants make over time. Being “more musical” may refer to the point in a person’s musical development where they can manipulate more parameters, for example, tone, dynamics and timing to find their own way of playing a phrase. This is a complex process which needs skill, time and experience to realize.

Service users also perceived their own musical skills improving:

When I first started I didn’t know how to play a guitar, but now I’m really good at it. [music staff] actually found me improving in how to play it, even though it’s a left handed one (Participant/G1/1).

This person appreciated a big change in what they were able to play, coming from being a complete beginner to being “good.” They also overcame an additional barrier in negotiating a left-handed guitar, which can be confusing when everyone else in the room is playing the standard right-handed instrument.

Lay researcher KB identified sophisticated musical skills in a service user who liked drumming:

he’s got such a good musical ear that he picks up the drumming, back to the drumming section again. He picks up almost immediately what (music staff) is doing (KB).

Different aspects of musicianship are expressed in this extract. Having a “musical ear” in this context demonstrates knowing the structural features of a song, i.e., what drumming pattern you are meant to play where. “Picking” up on the intentions of the music staff correctly requires good contextual knowledge of the song and happens quickly, often in a few words and gestures rather than step-by-step explanation or reading music.

Lay researcher MM identified the benefits of having music staff who also had a disability as making a positive contribution to an inclusive musical environment. He attributed this to Limelight Music creating an atmosphere where “it’s not them and us,” exemplified by having a role model, a musician with a disability. Another factor which may have influenced the strength of this effect for Group 1 is the lack of opportunity in music college courses or modules available in the area. One parent explained “college courses are all about life skills, about surviving, how to do what you need to do just now” (Parent/G1/9). The workshops provided her son with a chance to refine his music skills with experts. A final contributing factor is that some of Group 1 had been part of Stage 1 and so had a strong basis from which to extend existing musical skills.

#### Mixed Response

Staff, family and participants of Group 2 reported a *mixed response* to the workshop content. They all enjoyed the music staff’s use of humor: “sometimes it’s funny, because usually you get a bit of banter” (Participant/G2/2). They all enjoyed the social aspects of the workshops, but the musical activities were viewed in sharply different ways.

One participant expressed a wish to hear and play different music:

Participant: Aye. It’s the same songs every time.

Interviewer: What other songs would you like to do?

Participant: Pop music…. But fast music, rock and roll, Rod Stewart (Participant/G2/4).

One staff member offered an explanation for the service users’ feeling about the content: “If things get too repetitive they’re like, we’ve done it!” It is possible that the purpose of the workshops was not understood fully; the nature of development in music workshops to where change is comfortable for all may have felt too slow. If participants missed workshops then music staff would have to repeat songs so that the group had a cohesive understanding before moving to new material. In this way non-attendance can create a hiatus in the process of improvement in the music group and consequently repetition of known material could affect motivation to come back.

On the other hand, other participants in Group 2 were identified as benefiting from a known structure:

He likes that he knows when he goes in …what they’re going to be doing and he knows what’s coming next, he knows the words to the songs, he knows the instruments that they use and stuff like that. Obviously (music staff’s name) is there every week so he knows that that is who is going to be taking the class. So that’s important to him, so I think that is what works for him (Service staff/G2/5).

Balancing different needs and preferences presents a challenge for music staff. Groups 1 and 3 had very good attendance and all were able to contribute to and enjoyed playing in the group. This suggests other external factors outside of workshop content can impact on cohesiveness of a group and motivation to attend. Group 2 were presented with an alternative activity, arts and crafts, during the scheduled workshop time which six participants chose to pursue instead of the music sessions.

#### Lasting Positive Change in Mood

In Group 3, both center and residential staff, as well as families of the participants described a lasting positive change in mood as a key impact in both post-workshop and follow up interviews, 2 months after the workshop program was complete. This was noted strongly in over half of the center users (*n* = 8).

For one service user, Jack, his mum had reported that in the year leading up to the workshops she noticed him withdrawing and not being interested in activities that had previously given him joy. After months of investigation by medical and social care professionals, it was concluded that there was no obvious reason for this and perhaps he had depression. Through the workshops he “came back to life” (staff member) and started joining in with enthusiasm. Jack began making jokes during the workshop and at home, using the songs he learned in the workshops to play with expectations. For example, in the breakfast song, he claimed that he had very different and extravagant food than normal. His mum saw this as him “regaining sense of humor, even in a small way.” She said that staff reported that during the workshops:

it’s like seeing the old Jack. You know, wee windows. I don’t think for the whole time, because his concentration’s not … you’re never going to get that length of time. But they said they’ve just seen bits of him that’s like they used to see years ago, coming back. So they thought it was great. Because sometimes I don’t think they can get him to participate in very much (Parent/G3/1).

During the workshops, Jack would come home and put music on of his own volition, continuing this after the workshop program ended.

When we come home, as soon as he came in he puts music on in his room and he was singing away….he’d be quite enthused… it was all himself, self-motivated sort of thing, which is good (Parent/G3/1).

Housing staff for Group 3 described an ongoing positive mood for eight service users: “Laura herself has continued to have a very positive outlook” (Residential staff/G3/1), attributing this to their experience in the workshops that contributed to a growing resilience. For example, one participant who was in a married couple living in sheltered housing had dealt with her husband’s sickness better than expected: “some of that could be contributed to the fact she’s feeling quite positive about life generally at the moment, even though she is facing difficulty” (Residential staff/G3/2). Other participants said to be “really positive with the way things are in their lives” (Residential staff/G3/2) and this had an impact on previously “fractious” relationships with others in sheltered housing.

Staff identified the music workshops as providing a high quality and inclusive experience for their service users: “I think it’s something she can genuinely interact with and get something back from” (Service staff/G3/4). They compared the workshops favorably to other available activities, as they found that everyone in the group could join in some way:

Whereas music, she can genuinely contribute to that. Something can be put in her hand that she can shake if she wants to. If there was a vibration, if her hand was put on a speaker, she can feel all of that stuff. She can shout. I think it’s stress-relieving. All of that stuff. So, I think it’s something she can genuinely be involved in and it’s not just tokenism (Residential staff/G3/5).

Inclusiveness was enacted by music staff creating bespoke arrangements of songs for the group so individuals could contribute at a level that was appropriate for them.

#### Summary of Contextual Impacts

As well as impacts reported across all three groups, interviewees from each center described distinct outcomes from the workshops. Group 1 described how the musicianship of several members improved over the workshop program, as well as the group itself improving. Family members attributed this to the workshops addressing a gap in available education. As well as this, one of the music staff with disability provided a positive role model for the group. Potentially due to lower social cohesion, Group 2 reported mixed responses toward the music workshops, with some participants finding their structure too repetitive, while others enjoyed the predictability and familiarity of the activities. This led to some participants choosing to attend arts and crafts activities instead of the workshops, as they took place at the same time. Family and housing staff from Group 3 described them improving in mood and that this resulted in increases in self-initiated music listening and greater resilience. Participants found the workshop activities to be fun and engaging and they were able to contribute musically on different levels as suited their skills.

The next section reports the quantitative results on dimensions of change in social skills and the section following that reports the results from mixed methods analysis.

### Quantitative Results

There was a statistically significant increase in the following areas of the survey, comparing scores before and after the workshop program:

• Joint attention: *t*(14) = −3.389, *p* = 0.004, *M* = 3.60, and SD = 1.242 before the workshop program and *M* = 4.47, SD = 0.640 after the workshop program.
• Communication: *t*(14) = −3.523, *p* = 0.003, *M* = 3.33, and SD = 1.397 before the workshop program and *M* = 4.47, SD = 0.834 after the workshop program.
• Interaction with others: *t*(14) = −3.108, *p* = 0.008, *M* = 3.53, and SD = 1.302 before the workshop program and *M* = 4.47, SD = 0.915 after the workshop program.

There was no statistically significant increase found in self-expression or social awareness, as measured in the survey used.

### Mixed Methods Analysis

By combining qualitative and quantitative results, a deeper understanding of the identified themes from the interviews is possible, as well as where they overlap. As the survey was concerned with social development, the theme of mood was not topically relevant and there was no convergence of data for this theme. The following section expands on Table 2.

**TABLE 2:** Data convergence table.

Qualitative results (themes)	Quantitative results (statistically significant increase in these areas)	Alignment
Self-expression	Communication	Confirm
Confidence	Communication Interaction with others	Confirm
Social skills	Joint attention Communication Interaction with others	Confirm, enhance

#### Dimensions of Self-Expression

This theme explored the capacity of the participants in the following ways: ability to tell “my story” through creating or contributing to song lyrics; convey feelings through playing instruments or singing and communicate musical intent through non-verbal modes of communication (gaze, gesture, and movement). The survey element of communication *confirms* the qualitative data, as it was a measure of participants’ ability to form and articulate a request for themselves.

#### Dimensions of Confidence

The confidence theme was articulated as participants believing in their personal choices and being able to communicate these; coping better with uncertainty and change; and initiating communication with others. Survey elements of communication and interaction *confirm* this theme.

#### Dimensions of Social Skills

Improvements in social skills were described as participants having increased number of interactions with others and in more ways (e.g., verbal, eye contact, and moving to sit next to someone new) as well as their social circles expanding. Survey elements of joint attention, communication and interaction with others confirm this theme. Joint attention enhances this theme as interviews did not describe social skills in this way. The music workshops were a clear example of a shared focus which was perhaps taken for granted.

In summary, aspects of the qualitative results were confirmed and enriched by quantitative results. Combining results also demonstrated the overlapping relationships between themes, for example self-expression and confidence were confirmed by the quantitative element of communication. Social skills overlapped with both self-expression and confidence, with quantitative elements of communication and interaction with others.

## Discussion

Results confirmed that a community music program can benefit adults with disabilities, but evidenced through the use of innovative mixed methods, these impacts can vary for groups with different characteristics and needs. Common effects were positive impacts in self-expression, confidence, mood and social skills. These are related processes, for example, self-expression could be appreciated in different modes of communication in this inclusive workshop program, as participants had space to express themselves in their own way. The development of the personal qualities (self-expression, confidence) and improvements in wellbeing (mood) could be viewed in a range of social interactions (Bishop, 1998). As well as similar impacts, some differences between groups were found. Group 1 were identified as developing musical skills, Group 2 had a mixed experience and Group 3 reported a lasting positive effect on mood in 8 out of 11 participants. Appreciating how and why the same workshop program had varied effects is a key contribution of this paper. In particular, each group faced different social inequalities, for instance unequal access to music, resulting in different experiences of participation (Daykin, 2012). Gaining more understanding of the effects of community music and how they can be captured for different groups will allow benefits to be made accessible to this population as a whole, and reduce the wider inequalities in mental health.

Mixed methods were the most suitable way to appreciate the multiple and varied effects of this complex intervention for the diverse people taking part in the same workshop. The person-centered research design was qualitatively led. This had the benefit of gaining a greater understanding of the participants’ environments, such as challenges due to location, lack of opportunity or clash in activities, a crucial feature in research with those who have ASC (Bushell, 2017). Combining data from both qualitative and quantitative approaches allowed for triangulation of qualitative results and an enhancement of themes. Another benefit of a qualitative-led approach is to identify appropriate quantitative tools. As some impacts were common to more than one setting, additional methodological tools could be employed to capture more of these through quantitative approaches. For instance, a survey adapted from Profile of Mood States (McNair et al., 1992), the Intellectual Disability Mood Scale (Argus et al., 2004) could be used to capture dimensions of effect on mood.

Community music programs have potential to benefit the mental health and wellbeing of a broad range of people with disabilities, but importantly this research highlights that they can do so in different ways for particular groups and settings. Positive effects on different dimensions of mental health can be appreciated in addition to improvements in mood. For Group 1, improvements in musical skills created a growing feeling of competency and for some, a powerful feeling that music participation could change their life. The flexibility with which self and other are brought into being is seen as being at risk in individuals with ASC in particular but amenable to creative interventions (Bottema-Beutel et al., 2017), and this development of a musical identity through workshop participation is an example of a fundamental change for a person, as it is an additional channel for self-expression (MacDonald and Miell, 2002; Wilson and MacDonald, 2019). Group 1 were also able to gain the benefits of recognition for their musical skills from peers, music workers, staff, as well as their families. This group also had a performance to work toward which provided external motivation and recognition for their musicianship. This may have not benefited the other groups in the same way if they had taken part in such an event, as research shows that challenges (inherent in live performance) are best met when the person has positive beliefs about music and themselves (Lehmann et al., 2007). Group 1 continued their relationship with Limelight after the study finished, with service staff independently applying for funding to facilitate more workshops. This demonstrates positive engagement, but also that music interventions would benefit participants if on a longer-term basis. Future studies could usefully address this issue.

Participants in rural areas have more barriers, such as loneliness, lack of agency, and boredom due to fewer opportunities. There is a similar structure and activities in school and for school leavers in day centers. Reasons for this intervention being particularly beneficial for mood in Group 3, could be that it offered enough stability to create a secure environment and sufficient change to be engaging. Taking part in music groups has been proposed as providing a positive coping strategy for those with a disability (Kerr et al., 2012), an assertion which is supported by this study. Future research could usefully focus on investigating effects on participants’ coping strategies and resilience in more detail.

Research Principles in Person-Centered Research (Jacobs et al., 2017) Informed This Study the Following Ways:

### Connectivity

This study had many stakeholders who were able to contribute their views through different channels. These were an advisory group with monthly meetings, and open public discussion during the showcase event at the end of the project. Two lay researchers who have a disability, KB and MM, contributed their thoughts on a draft of this paper, resulting in further conceptual development of themes, in particular, KB’s description of a participant’s “voice” being expressed in non-verbal modes of communication.

### Attentiveness and Dialog

The research team made several visits to all centers to both observe and participate in the workshops. An important part of these visits was for service users to meet the researchers as “a relaxed, informal interaction …with trust and rapport” are crucial when interviewing individuals with a disability (Prosser and Bromley, 2012, p109).

### Empowerment and Participation

Participants had agency within the music workshops, they were able to choose whether to attend, how to join in (or not), what instruments they wanted to play, and their ideas became part of songs which formed the group’s repertoire. Before every workshop, verbal assent was sought and six participants from Group 2 chose to attend art instead. One participant was very gifted in this discipline and was perhaps more motivated to participate in an activity in which he had more mastery. Interestingly, the participants who dropped out were from one of the two subgroups comprising Group 2 and so may have demonstrated a social cohesiveness of their own choosing, by taking part in art classes together.

### Critical Reflexivity

In taking a person-centered approach, the rights and views of the participants were prioritized throughout this study. This is in line with demands for a commitment and focus among non-disabled researchers toward genuine engagement, rethinking research relationships, inclusive design, and an empowered voice for people with intellectual disabilities (Watchman et al., 2020). It has benefited the research through development of methodological tools and integration of analysis. Methods and implementation of these were adapted to priorities the research participants. All participants were invited to be interviewed and could do so with or without support (either from service staff or a lay researcher). The quantitative measure was co-designed with service staff based on measures of social development already in use.

## Conclusion

Developing holistic research projects that are ecologically valid, including qualitative and quantitive aspects and involving service users and stakeholders, is of paramount importance to increase the impact and relevance of academic research. This study apprehended and measured the complex impacts of a community music intervention through a mixed-methods, qualitatively led person-centered approach. This approach adds to the small but growing body of mixed methods research in community music (e.g., Fitzpatrick, 2011; Grocke et al., 2014), particularly by incorporating a person-centered approach. It has contributed to calls for greater understanding of, and sensitivity to, the environment of participants with a disability (Bushell, 2017), with mixed methods approaches in naturalistic settings seen as key to ensuring this sensitivity in research (Hochhauser and Grynszpan, 2017; Vann and Gasson, 2017). The lay researchers provided novel and invaluable perspectives in analysis of results, firstly in outlining the multimodal nature of self-expression for those with a disability and secondly in identifying the importance of having a positive role model among the music staff who was a skilled musician with a disability.

This research has identified positive effects of music participation on aspects of mental health in terms of self-expression, confidence, mood, social skills, communication skills, joint attention and interaction with others, which align with previous work (Kerr et al., 2012; Leadbetter and O’Connor, 2013; All-Party Parliamentary Group on Arts and Health and Wellbeing, 2017). This has great utility, as mental health has been identified as a key area of wider health inequalities that people with disabilities face (Honey et al., 2011). The inclusive approach to community music projects reported here, which feature differentiated activities, tailored to meet the individual needs of participants and the social and cultural context in which they were living, offered a unique creative activity and separate channel for self-expression and interaction. This research has also shown the nature of engagement in community music workshops to be mediated by environment and experience; activities should be tailored to accommodate this aspect. A preliminary phase to future workshops in collaboration with lay researchers could investigate values, beliefs and experiences of participants to maximize what they can gain from workshops. In this way, positive coping strategies can develop for individuals with a disability through an engaging, absorbing and rewarding experience of music, and add to international commitments to improving health and wellbeing for this population.

## Data Availability Statement

The datasets for this study will not be made publicly available because the terms of consent for research participation did not specify that data would be accessible other than by the research team.

## Ethics Statement

This research received ethical approval from the Edinburgh College of Art Research Ethics Sub-Committee, University of Edinburgh, which includes consideration of any risk of disadvantaging individuals through the research. Informed consent for research participation was gathered in advance from participants, or from an identified person with responsibility if they lacked capacity for this. To inform potential interviewees, accessible printed or recorded information sheets were prepared detailing the voluntary, confidential and anonymous nature of participation and how data would be used.

## Author Contributions

All authors listed have made a substantial, direct and intellectual contribution to the work, and approved it for publication.

## Conflict of Interest

The authors declare that the research was conducted in the absence of any commercial or financial relationships that could be construed as a potential conflict of interest.

## Footnotes

1 Lay researchers were Kirsty Brown, henceforth (KB) and Michael McEwan, henceforth (MM).

2 Interviewees will be described by data set (e.g., Service staff, Participant) Group number (1-3) and ID number within the group.

3 The Year of Young People was part of the Scottish Government’s themed-years program. It was a co-designed collaborative project aiming to provide a platform for young people to have their views heard and acted upon. https://www.webarchive.org.uk/wayback/archive/20190923175906/http:/yoyp2018.scot/opportunities/.
